# The pitfalls associated with urinary steroid metabolite ratios in children undergoing investigations for suspected disorders of steroid synthesis

**DOI:** 10.1186/s13633-015-0007-1

**Published:** 2015-04-15

**Authors:** Angela K Lucas-Herald, Martina Rodie, Laura Lucaccioni, David Shapiro, Jane McNeilly, M Guftar Shaikh, S Faisal Ahmed

**Affiliations:** Developmental Endocrinology Research Group, Royal Hospital for Sick Children Yorkhill, Dalnair Street, Glasgow, G3 8SJ UK; Department of Biochemistry, Glasgow Royal Infirmary, Alexandra Parade, Glasgow, G4 0SF UK; Department of Biochemistry, Southern General Hospital, Glasgow, G51 4TF UK

**Keywords:** Urine, Disorders of sex development

## Abstract

**Background:**

Urinary steroid metabolite ratios may improve the diagnostic yield of potential disorders of steroid hormone synthesis.

**Objectives:**

To investigate the range of ratios and their predictive value in children with suspected disorders of steroid synthesis.

**Design and methods:**

Twelve ratios were calculated on steroid metabolite data analysed by gas chromatography–mass spectrometry in urine samples collected between 2008–2010 from 93 children. Urine samples were also analysed in 252 children with no known endocrine concerns.

**Results:**

Of the 252 controls, 115 (46%) were male with a median age of 10 yr (range 1 month,18.5 years). Of the 93 cases, 38 (41%) were male with a median age of 6.5 yr (1 day,18.5 yrs). Of these, 41 (44%) had at least one ratio greater than the 95% percentile for controls. The most frequently abnormal ratio, found in 18/93 (19%) cases was (THS/(THE + THF + 5αTHF)) suggestive of 11β-hydroxylase deficiency. Over this period, 8 (9%) children were subsequently diagnosed with a steroid hormone disorder; 4 with 21-hydroxylase deficiency, 2 with11β-hydroxylase deficiency and 2 with 5α-reductase deficiency. All except one of these children had at least 1 raised ratio.

**Conclusions:**

Urinary steroid metabolite ratios in suspected disorders of hormone synthesis often exceed the reference range for normal children. The predictive value of steroid metabolite ratios in identifying a genetic abnormality may be condition specific and needs further study to improve its clinical utility.

## Introduction

Assessment of urinary steroid metabolite excretion is reported to be a valuable tool in the investigation of disorders of steroid hormone synthesis as it offers the opportunity to simultaneously examine many different steroids [1,2]. It is non-invasive, avoids the need for multiple blood tests and may provide reliable information on spot urine samples.

The standard method of analysing urinary steroid metabolites involves gas chromatography/mass spectrometry (GC-MS) [3]. Given that individual metabolite concentrations will be altered depending on the level of the enzyme block, the calculation of a urinary steroid metabolite ratio (uSMR) using GC-MS steroid profile results may be a particularly valuable method of improving diagnostic yield when investigating such disorders [4,5]. However, reference ranges for these ratios are not widely available and it is unclear whether such ratios need to be age and sex matched. In addition, as individual biosynthetic disorders may be associated with an abnormality of multiple ratios the clinical utility of calculating ratios in the diagnostic pathway remains unclear.

The aims of this study were, therefore, to determine reference ranges for a range of urinary steroid ratios and to explore the clinical utility of such ratios in a typical childhood population under investigation for suspected disorders of steroid synthesis.

## Subjects & methods

Of a total of 127 urinary steroid profiles collected between 2008 and 2010 at a single children’s hospital, 31 (24%) cases were excluded as they were 19 years old or over, were undergoing a dexamethasone suppression test or a synacthen stimulation test or were on steroid replacement therapy. Of the 96 eligible cases, sufficient clinical information was unavailable in 3. Of the remaining 93 cases, 38 (41%) were male and the median age at the time of the test was 6.5 years (range 1 day, 18.5 years). A total of 17 cases (18%) were <6 months at the time of test; 60 (65%) were 6 months-10 years of age and 16 (17%) were >10 years of age at the time of test.

A review of the clinical records was conducted for all cases to confirm the clinical characteristics, diagnosis and outcome. Of the 93 cases, indications for assessment of urinary steroid profile included early signs of puberty in 21 (22%), adrenarche in 16 (17%), suspected disorder of sex development in 10 (11%), congenital adrenal hyperplasia in 8 (9%) and Cushing’s syndrome in 2 (2%). In the remaining 36 (39%) cases, no clear reason for the test was available in the clinical records.

Urine samples from two groups of children were collected to develop a reference range. The first group consisted of 96 children with no background of endocrine concerns who had a urine sample collected at presentation for investigation of an acute systemic illness. Eight of these patients were excluded as they had a very high urine free cortisol concentration >1000 nmol/l at the time of urine sample. Urine samples were also collected from 164 healthy school-age children. Of a total of 252 controls, 115 (46%) were male and the median age at sample collection was 10 years (range 1 month-18.5 years). The controls were broadly categorised into three age bands <6 months, 6 months to 10 years and more than 10 years.

The urinary steroid metabolite analysis was performed by gas chromatography/mass spectrometry (GC/MS) using the Thermo Scientific Trace Gas Chromatography machine attached to an ITQ900 mass spectrometer. The method for analysis was a modification of the method originally described by Shackleton [6].

Twelve ratios as listed in Table 1 were calculated for each case and control [5,6]. Table 2 demonstrates the type of enzyme defect suggested by an abnormal ratio. If samples had not been reported due to a technical difficulty during analysis or there was insufficient sample for analysis and there was a lack of numerical data to apply a ratio, they were excluded from analysis. Table 1 demonstrates the number of cases analysed per ratio. Medians and ranges were determined using Microsoft Excel 2010 for all patients and controls. Kruskal-Wallis tests were performed for all samples using SPSS v 21 (IBM, 2013). Significance was determined if p ≤ 0.05.

**Table 1:** **Urinary steroid ratios and the number of cases with an abnormal ratio**

**Ratio**	**Cases analysed out of 93**	**Cases >95** ^**th**^ **percentile**	**Cases of suspected steroid disorder with ratio >95** ^**th**^ **percentile (% of those with ratio >95** ^**th**^ **percentile)**	**Sensitivity**	**Specificity**	**Positive predictive value**	**Negative predictive value**
17HP/(THE + THF + 5αTHF)	93 (100%)	18	4 (22%)	100%	84%	22%	100%
PT/(THE + THF + 5αTHF)	92 (99%)	16	4 (25%)	100%	86%	25%	100%
PTONE/(THE + THF + 5αTHF)	61 (65%)	11	5 (45%)	100%	89%	45%	100%
THS/(THE + THF + 5αTHF)	92 (99%)	23	4(17%)	100%	78%	17%	100%
DHEA/(THE + THF + 5αTHF)	90 (96%)	12	2 (17%)	100%	90%	17%	100%
(17HP + PT)/(An + Et)	85 (90%)	6	2 (33%)	100%	95%	33%	100%
(THF + 5αTHF)/THE	93 (100%)	7	1 (14%)	100%	93%	14%	100%
THE/(THF + 5αTHF)	93 (100%)	9	1 (11%)	100%	91%	11%	100%
(An + Et)/(THE + THF + 5αTHF)	89 (95%)	6	1 (17%)	100%	94%	17%	100%
Et/An	84 (89%)	6	2 (33%)	100%	95%	33%	100%
THF/5αTHF	93 (100%)	10	2 (20%)	100%	91%	20%	100%
THB/5αTHB	86 (91%)	6	1 (17%)	100%	94%	17%	100%

17HP: 17-hydroxypregnanolone, PT: pregnanetriol, THE: tetrahydrocortisone, THF: tetrahydrocortisol. 5αTHF: 5a-tetrahydrocortisol. An:androsterone. Et: etiocholanolone. DHEA: dehydroepiandrosteroone. PTONE: pregnanetriolone. THS: tetrahydro-11-deoxycortisol. THS: tetrahydro-11-deoxycortisol. THB: tetrahydrocorticosterone. 5αTHB: 5α-tetrahydrocorticosterone.

**Table 2:** **Median, range and 95** ^**th**^ **centile for each urinary steroid metabolite ratio analysed for the whole group of controls as well as categorised by broad age groups**

		**All controls N = 252**	**<6 months N = 12**	**6 months-10 yrs N = 106**	**>10 yrs N = 134**
Enzyme defect	Median age	10 years	2 months	5 years	13 years
	(Range)	(11d-18 yrs)	(11 d-5 m)	(6 m-9 yrs)	(10–18 yrs)
	M:F ratio	115:137	7:5	47:59	61:73
21-Hydroxylase def	**17HP/(THE + THF + 5αTHF)**				
	Median (range)	0.01 (0–0.25)	0.01 (0.01-0.11)	0.01 (0–0.25)	0.01 (0–0.11)
	95% centile	0.04	0.1	0.03	0.04
21-Hydroxylase def	**PT/(THE + THF + 5αTHF)**				
	Median (range)	0.03 (0–0.48)	0.02 (0.01-0.03)	0.02 (0–0.28)	0.06 (0.01-0.48)
	95% centile	0.13	0.03	0.09	0.15
21-Hydroxylase def	**PTONE/(THE + THF + 5αTHF)**				
	Median (range)	0 (0–0.29)	0.01 (0–0.14)	0 (0–0.29)	0 (0–0.04)
	95% centile	0.01	0.11	0.02	0.01
11β-Hydroxylase def	**THS/(THE + THF + 5αTHF)**				
	Median (range)	0.01 (0–0.09)	0 (0–0.04)	0.01 (0–0.09)	0 (0–0.02)
	95% centile	0.02	0.03	0.03	0.01
3β-Hydroxysteroid dehydrogenase def	**DHEA/(THE + THF + 5αTHF)**				
	Median (range)	0 (0–1.25)	0.01 (0–0.1)	0 (0–1.25)	0 (0–0.31)
	95% centile	0.12	0.1	0.03	0.14
P450 oxidoreductase def	**(17HP + PT)/(An + Et)**				
	Median (range)	0.38 (0.1-12.5)	1.95 (0.5-5)	0.75 (0.17-12.5)	0.23 (0.1-0.75)
	95% centile	3.18	4.52	3.93	0.46
Apparent mineralocorticoid excess	**(THF + 5αTHF)/THE**				
	Median (range)	0.72 (0.01-7)	0.04 (0.01-0.35)	0.8 (0.15-7)	0.68 (0.25-.3.43)
	95% centile	1.97	0.33	3.01	1.3
Apparent cortisone reductase deficiency	**THE/(THF + 5αTHF)**				
	Median (range)	1.4 (0.16-88.69)	22.82 (2.87-88.69)	1.25 (0.16-20.9)	1.49 (0.29-20.7)
	95% centile	3.84	86.58	2.39	3
17β-Hydroxysteroid dehydrogenase deficiency	**(An + Et)/(THE + THF + 5αTHF)**				
	Median (range)	0.11 (0–1.65)	0.01 (0.01-0.09)	0.03 (0–0.75)	0.3 (0.04-1.65)
	95% centile	0.7	0.07	0.23	0.9
5α-Reductase deficiency	**THF/5αTHF**				
	Median (range)	0.73 (0.08-25.25)	0.96 (0.19-5)	0.68 (0.08-25.25)	0.83 (0.22-2)
	95% centile	1.75	4.19	1.93	1.71
5α-Reductase deficiency	**THB/5αTHB**				
	Median (range)	0.28 (0–68)	20.1 (0.33-68)	0.27 (0.02-47.64)	0.39 (0–2.05)
	95% centile	3.89	53.06	1.97	1.24
5α-Reductase deficiency	**Et/An**				
	Median (range)	0.59 (0.03-4.86)	0.13 (0.03-1)	0.56 (0.06-4.86)	0.64 (0.26-2.1)
	95% centile	1.7	0.69	2	1.5

17HP: 17-hydroxypregnanolone, PT: pregnanetriol, THE: tetrahydrocortisone, THF: tetrahydrocortisol. 5αTHF: 5α-tetrahydrocortisol. An: androsterone. Et: etiocholanolone. DHEA: dehydroepiandrosteroone. PTONE: pregnanetriolone. THS: tetrahydro-11-deoxycortisol. THB: tetrahydrocorticosterone. 5αTHB: 5α-tetrahydrocorticosterone.

The urine samples from the healthy school children were collected following informed consent as part of a previous study of growth and development that was approved by the local research ethics service. The additional samples that were collected from children attending hospital were approved by the ethics service as part of local assay development.

## Results

Table 2 demonstrates reference ranges for each of the ratios. Analysis was performed to determine whether age or gender influenced the results of the results of the ratios. No significant difference was found between the urinary steroid metabolite ratios when categorised by age and sex.

Of the 93 cases, 53 (57%) had ratios that were greater than the 95% percentile for controls (Table 1). The number of ratios per case which were greater than the 95% percentile for controls ranged from 0–8 (median 1). Of these 53 cases with high ratios, 25 (47%), 10 (19%), 7 (13%) and 11 (21%) children had only 1, 2, 3 or >3 raised ratios, respectively (Table 1).

The most commonly abnormal ratio when cases were separated according to gender and compared with controls was THS/(THE + THF + 5αTHF), with 20 of 53 (38%) cases having ratios greater than the 95% percentile for controls. The most commonly abnormal ratio when cases were separated according to age and compared to controls was DHEA/(THE + THF + 5αTHF) with 19 of 53 (36%) cases. When cases were not split for either age or gender, the most commonly abnormal ratio was THS/(THE + THF + 5αTHF) which was greater than the 95% percentile for controls in 24 of 93 (26%) cases.

**Figure 1:**
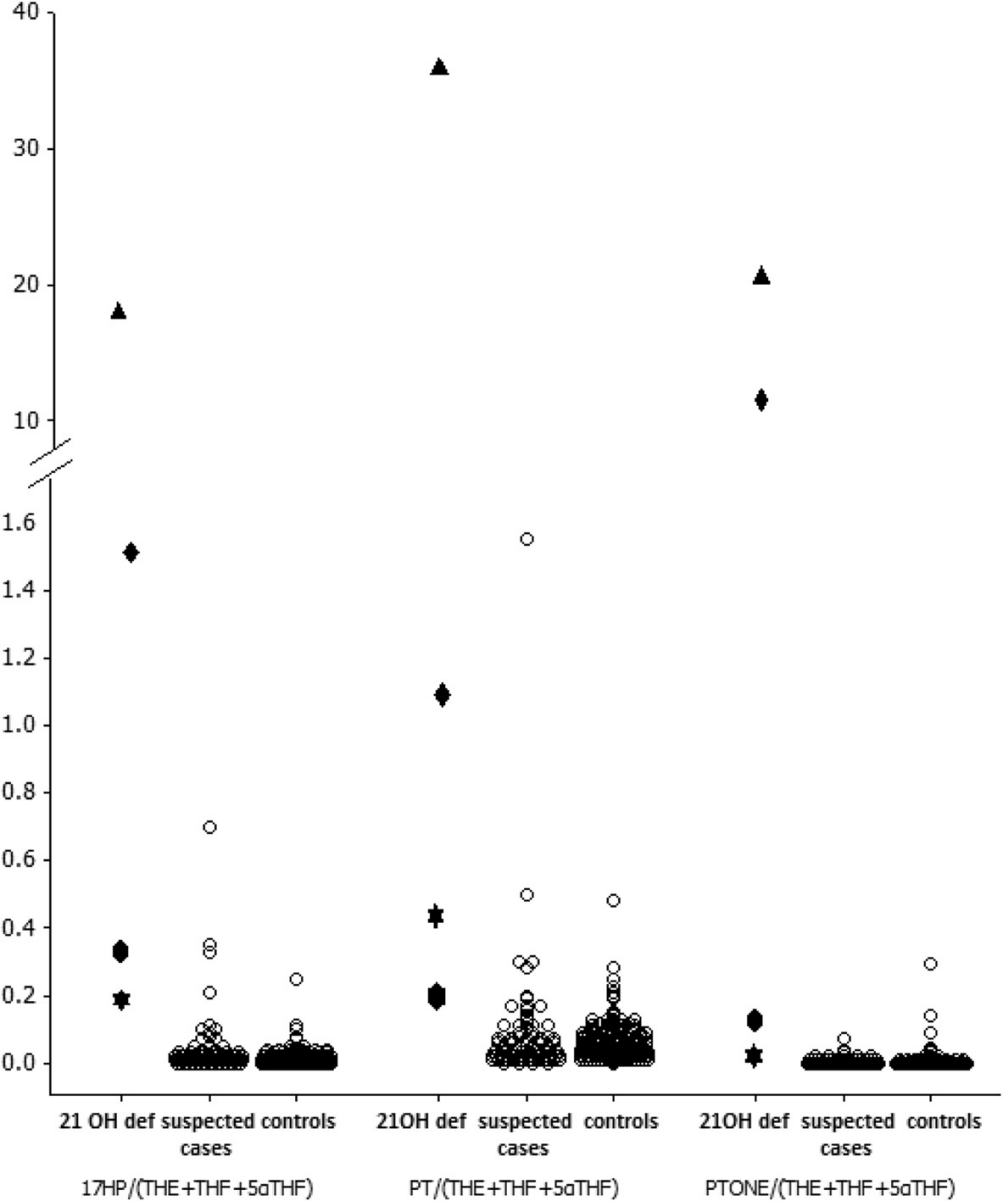
**Urine steroid metabolite ratios for investigating 21-Hydroxylase deficiency.** Ratios for confirmed cases of 21-hydroxylase deficiency, possible cases and controls. Each confirmed case is a different shape to demonstrate results of different ratios.

A total of 8 cases were subsequently diagnosed with a steroid hormone disorder (true positives); 4 (44%) with 21-hydroxylase deficiency, 2 (22%) with 11β-hydroxylase deficiency and 2 (22%) with 5α-reductase deficiency (Table 3). All except one of these cases had at least 1 ratio greater than the 95% percentile for controls. The case with the normal steroid hormone ratios was later diagnosed with 5α-reductase deficiency. The median number of abnormal ratios in the 4 cases with 21-hydroxylase deficiency was 4.5 (range 4–6). All of these cases had ratios greater than the 95% percentile for controls for the 3 ratios suggestive of 21-hydroxylase deficiency Figure 1). Three of these patients had genetic confirmation of CYP21A2 mutations. The other patient had been given this diagnosis secondary to suggestive biochemistry results and clinical history. Genetic analysis had not been performed for patients with any of the other disorders of steroid synthesis.

**Table 3:** **Characteristics of confirmed cases**

**Sex**	**Age**	**Presenting features**	**Biochemistry**	**Ratios >95** ^**th**^ **percentile**	**Actual ratio**	**Diagnosis**
M	5y	Subclinical salt wasting	Testosterone 5.1	17HP/(THE + THF + 5αTHF)	1.5	21-hydroxylase deficiency
		Tall stature and sexual precocity	17 OHP: 443	PT/(THE + THF + 5αTHF)	1.1	CYP21A2 mutation
			Androstenedione NA	PTONE/(THE + THF + 5αTHF)	11.5	
				THS/(THE + THF + 5αTHF)	0.04	
				DHEA/(THE + THF + 5αTHF)	0.14	
				(17HP + PT)/(An + Et)	15.1	
				(THF + 5αTHF)/THE	16.8	
				THB/5αTHB	5.3	
M	10d	Salt wasting presented with vomiting and weight loss.	17 OHP: NA	17HP/(THE + THF + 5αTHF)	0.2	21-hydroxylase deficiency
			Androstenedione NA	PT/(THE + THF + 5αTHF)	0.5	CYP21A2 mutation
				PTONE/(THE + THF + 5αTHF)	0.1	
				THE/(THF + 5αTHF)	62.3	
F	13y	Presented at 8 yrs with exaggerated adrenarche. Not salt wasting	17 OHP: 118	17HP/(THE + THF + 5αTHF)	0.3	21-hydroxylase deficiency
			Androstenedione 11	PT/(THE + THF + 5αTHF)	0.2	
				PTONE/(THE + THF + 5αTHF)	0.1	
				DHEA/(THE + THF + 5αTHF)	0.7	
F	3y	Clitoromegaly with no other signs of virilisation. Not salt wasting	17 OHP 80	17HP/(THE + THF + 5αTHF)	18.6	21-hydroxylase deficiency
			Androstenedione 30	PT/(THE + THF + 5αTHF)	37.2	CYP21A2 mutation
				PTONE/(THE + THF + 5αTHF)	21.6	
				(17HP + PT)/(An + Et)	36.7	
				(An + Et)/(THE + THF + 5αTHF)	1.5	
F	11y	Premature adrenarche. On steroid replacement. Hypertensive.	17 OHP 3	THS/(THE + THF + 5αTHF)	0.1	11β-hydroxylase deficiency
			Androstenedione 13.3			
			DOC – NA			
F	2 m	Clitoromegaly. Hypertensive.	17 OHP: 3	THS/(THE + THF + 5αTHF)	0.6	11β-hydroxylase deficiency
			Androstenedione < 1.4	PTONE/(THE + THF + 5αTHF)	0.02	
M	8y	Bilateral undescended testes, micropenis.	DHT < 0.1	Nil		5α − reductase deficiency
		Parents consanguineous	Androstenedione 1.5			
			Testosterone 0.7			
M	5y	Glandular hypospadias.	DHT < 0.1	THS/(THE + THF + 5αTHF)	0.04	5α − reductase deficiency
		Parents consanguineous.	Androstenedione 1.4	Et/An	5.1	
			Testosterone < 0.5	THF/5αTHF	31.8	

USMR: Urinary steroid metabolite ratio. 17HP: 17-hydroxypregnanolone, PT: pregnanetriol, THE: tetrahydrocortisone, THF: tetrahydrocortisol. 5αTHF: 5α-tetrahydrocortisol. An:androsterone. Et: etiocholanolone. DHEA: dehydroepiandrosteroone. PTONE: pregnanetriolone. THS: tetrahydro-11-deoxycortisol. THB: tetrahydrocorticosterone. 5αTHB: 5α-tetrahydrocorticosterone.

**Figure 2:**
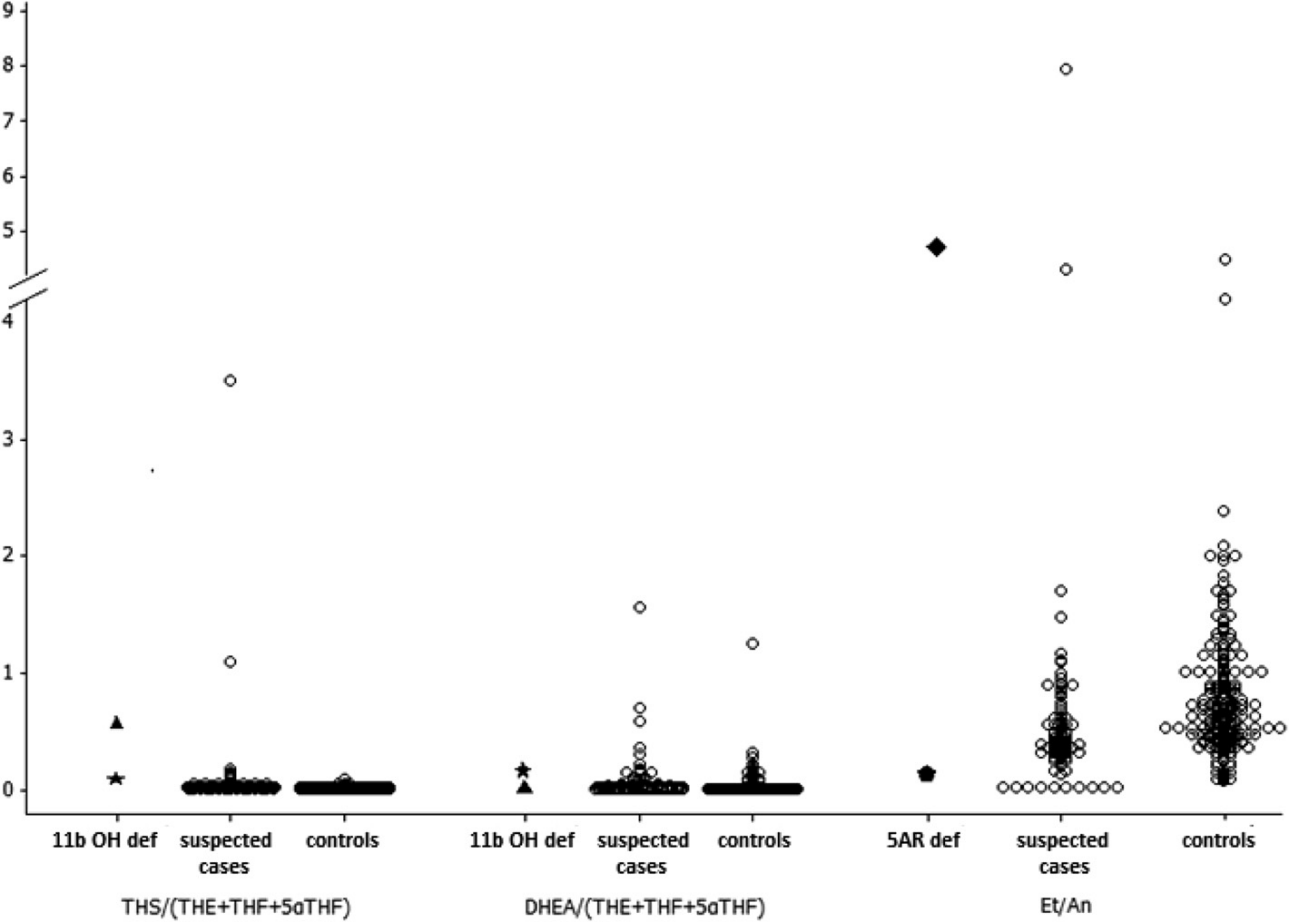
**Urine steroid metabolite ratios for investigating 11β-hydroxylase deficiency and 5α − reductase deficiency.** Ratios for confirmed cases of 11β-hydroxylase deficiency and 5α − reductase deficiency, possible cases and controls.

The two cases of 11β-hydroxylase deficiency both had a raised THS/(THE + THF + 5αTHF) ratio, which is suggestive of 11β-hydroxylase deficiency (Figure 2). One of these cases presented at 4 years with signs of premature adrenarche and had a repeat urine sample at the age of 11 years. This patient had only one raised ratio. The other presented with clitoromegaly at birth and also had a raised PTONE/(THE + THF + 5αTHF) ratio.

The 2 boys with 5α-reductase deficiency were 46,XY brothers who presented with atypical genitalia. They had consanguineous parents and were lost to follow-up before genetic investigations could be undertaken. One of them had 3 abnormal ratios (THS/(THE + THF + 5αTHF), suggestive of 11β-hydroxylase deficiency and Et/An and THF/5αTHF suggestive of 5α-reductase deficiency) and presented with glandular hypospadias and micropenis (Figure 2). His older brother who was also affected had no abnormal urinary steroid ratios and presented with bilateral undescended testes and micropenis.

## Discussion

Urinary steroid profiling is frequently performed in the investigation of patients with suspected disorders of steroid synthesis [7]. The clinical indication for this test has also widened over the last few years. Steroid profiling of maternal urine may be used as a method of prenatal detection of conditions such as Smith Lemli Opitz syndrome [8] and congenital adrenal hyperplasia caused by P450 oxidoreductase deficiency (PORD), with results suggesting that diagnostic steroid ratios could demonstrate PORD as early as at 12 weeks gestation [9]. Another recent study reviewed the use of urinary steroid ratios in the differential diagnosis of PORD and classic 21-hydroxylase deficiency in Japanese children, finding that the ratios were a reliable method of distinguishing these 2 conditions [10]. The calculation of simple ratios is thought to further improve the sensitivity and specificity of the steroid profile in the detection of disorders of steroid synthesis [5].

Our study provides references ranges for 12 steroid ratios that have been highlighted as the ratios which may be abnormally high in disorders of steroid synthesis [5]. The reference range was created from urine samples collected from a range of sources and we have clearly shown wide variations in urine steroid ratios in these reference data. It is possible that some of these variations may be due to differences in the age and sex of the child [11]. However, our data do not show a clear association to these variables when assessing steroid metabolite ratios indicating that these variations may only apply to absolute values. It is possible that if the age categories were over narrower age bands during childhood, then some age and sex related differences in ratios as reported before [12] may have been observed. It is also possible that some of the children from whom samples were obtained for creating the reference range had presented with an acute illness at the time of the urine sample and may have had raised steroid profiles secondary to these illnesses. To mitigate this possibility, those cases with very high urine cortisol excretion were, therefore, excluded. We have reported all the urine steroid ratios in early infancy for completeness but we should highlight that ratios for conditions such as 5α reductase deficiency and apparent cortisone reductase deficiency are difficult to interpret in early infancy.

We identified 8 children with a disorder of steroid hormone synthesis. A total of 7 of these cases had ratios greater than the 95% percentile for controls. Four of these cases had confirmed 21-hydroxylase deficiency and each of them had elevated ratios for the 3 ratios suggestive of this condition. Only 1 other case in this study, who had a sample collected at 18 days of age and was born at 28 weeks gestation, had this combination of 3 ratios which were greater than the 95% percentile for controls. For comparison, 6 other cases had ratios which were greater than the 95% percentile for controls for 17HP/(THE + THF + 5αTHF) and PT/(THE + THF + 5αTHF) alone. It is, therefore, possible that for 21-hydroxylase deficiency, genetic analysis could be routinely considered in those cases where all 3 ratios for 21-hydroxylase deficiency are raised. Depending on the availability of local resources, an alternative strategy would be to perform genetic analysis first and then pursue urine steroid biochemistry in those cases which do not reveal a gene mutation in CYP21A2.

Given that close to half of the cases had at least one abnormal ratio and 19% had two or more abnormal ratios, there is a need to clearly determine cut-offs regarding the number of ratios that need to be abnormal or the extent of abnormality that will prompt the need for genetic analysis. The likelihood of detecting a gene mutation, for instance in the CYP21A2 may be higher if all three ratios are raised to a very high level but published data supporting this, and its corollary, i.e. a low likelihood of a gene mutation in cases where not all ratios are mildly raised, are lacking. Our data also suggest that identification of pathology should not solely rely on steroid metabolite ratios. It is possible that a particular change in a ratio could occur due to different combinations of changes in its numerator and denominator. It would, therefore, be important to also pay attention to the absolute levels of the urine metabolites.

## Conclusions

In summary, several children who are investigated for a disorder of steroid synthesis have urinary steroid metabolite ratios that are above the reference range for normal healthy children. Some ratios are more specific than others for identifying disorders of androgen excess such as 21-hydroxylase deficiency. The predictive value of single and multiple steroid metabolite ratios in identifying a genetic abnormality needs further exploration.
